# Research trends and hotspots in publications jointly addressing remimazolam and midazolam: A bibliometric analysis, 2016–2025

**DOI:** 10.1097/MD.0000000000051418

**Published:** 2026-10-30

**Authors:** Yu Liu, Liang Cong, Shengzhen Luo, Yuying Ma, Hui Xiao, Yongwang Li

**Affiliations:** a Department of Anesthesiology, The Third People’s Hospital of Longgang, Clinical Institute of Shantou University Medical College (The Third People’s Hospital of Longgang District Shenzhen), Guangdong, China; b First School of Clinical Medicine, Guangdong Pharmaceutical University, Guangdong, China.

**Keywords:** anesthesia, bibliometric analysis, midazolam, remimazolam, research trends, sedation

## Abstract

**Background:**

This bibliometric study characterized the research landscape of literature jointly addressing remimazolam and midazolam from January 2016 through September 2025, with emphasis on publication trends, collaboration patterns, intellectual structure, and emerging research topics.

**Methods:**

Records published from January 1, 2016 through September 30, 2025 were retrieved from the Web of Science Core Collection on October 30, 2025 using a predefined topic search designed to identify publications in which remimazolam and midazolam were jointly represented. After eligibility screening, 349 publications were analyzed using CiteSpace, VOSviewer, and Bibliometrix to evaluate publication trends, collaboration networks, co-citation patterns, and keyword evolution.

**Results:**

Publication output increased markedly over the study period. China contributed the largest number of publications, followed by Japan, South Korea, and the United States. Major research themes included pharmacokinetics, safety, efficacy, and procedural sedation. Recent keyword signals included flumazenil, risk, and upper gastrointestinal endoscopy. Research productivity was concentrated within a limited number of journals and institutions, whereas international co-authorship was limited in this dataset.

**Conclusion:**

Research jointly addressing remimazolam and midazolam expanded markedly during the study period and was geographically concentrated, with prominent attention to pharmacokinetics, safety, efficacy, and procedural sedation. These findings provide a structured overview of how the field has evolved and highlight priorities for multinational collaboration, direct comparative studies, and patient-specific dosing research.

## 1. Introduction

Remimazolam is an ultra-short-acting benzodiazepine that produces sedative, hypnotic, and anxiolytic effects through positive allosteric modulation of γ-aminobutyric acid type A receptor signaling.^[1,2]^ It is characterized by rapid esterase-mediated metabolism to an inactive metabolite and a short, controllable pharmacokinetic profile.^[1–3]^ Its pharmacokinetics have also been specifically evaluated in subjects with hepatic or renal impairment.^[4]^ Like other benzodiazepines, its sedative effects can be antagonized with flumazenil.^[3]^

Midazolam is a widely used short-acting benzodiazepine and has served as an established comparator in clinical studies of remimazolam, including procedural sedation for colonoscopy and bronchoscopy.^[5,6]^ These direct comparisons, together with expanding studies of remimazolam across anesthesia and sedation settings, have created a rapidly growing body of literature in which the 2 agents are jointly discussed (Table 1).

**Table 1:** Bibliometric indicators of the top 10 authors ranked by h-index in the included remimazolam-midazolam literature.

Author	h_index	g_index	m_index	TC	NP	PY_start
Stoehr T	11	15	1.57	414	15	2020
Schippers F	10	10	1.43	586	10	2019
Pesic M	7	8	1.17	162	8	2020
Goudra B	5	6	0.83	102	6	2020
Petersen Ku	5	6	0.71	221	6	2019
Tobias Jd	5	7	1.25	61	8	2022
Zhang L	5	6	1.25	125	6	2022
Zhang Y	5	9	1.00	195	9	2021
Chen J	4	4	0.80	210	4	2021
Ichijima R	4	5	1	33	7	2022

NP = number of publications, PY_start = start year of publication, TC = total citations.

Since the introduction of remimazolam, research has expanded from early pharmacological characterization to comparative trials and diverse clinical applications.^[5–8]^ However, the development of this literature has not been fully characterized at the macro level. In particular, the evolution of publication output, collaborative networks, influential sources, co-cited references, and emerging research topics warrants systematic mapping to clarify how the field has developed and where major knowledge gaps remain.

Bibliometrics, an interdisciplinary approach integrating mathematical and statistical methods, enables systematic and quantitative analysis of scholarly publications.^[9,10]^ It can map the landscape of a research field by examining the publication volumes, citation networks, collaboration patterns, keyword co-occurrence, and thematic evolution. Visualization tools such as CiteSpace^[11,12]^ and VOSviewer^[13,14]^ facilitate the generation of knowledge maps, effectively delineating research frontiers and intellectual bases. Compared to traditional narrative reviews, bibliometric analysis provides a more objective, comprehensive, and dynamic perspective on disciplinary progress.

This study, therefore, used bibliometric methods to characterize the research landscape of publications jointly addressing remimazolam and midazolam from January 2016 through September 2025. The objectives were to quantify publication output, identify leading countries, institutions, authors, and journals, map collaboration and co-citation networks, and detect evolving keywords and emerging research topics. By delineating the structure, development, and thematic evolution of this literature, the study aims to provide a comprehensive overview of the field and identify priorities for future comparative and clinical research.

## 2. Methods

### 2.1. Data source and search strategy

The Web of Science Core Collection (WoSCC) was used as the sole bibliographic data source to maintain a consistent citation-data structure and because it is widely used in biomedical bibliometric analyses.^[15,16]^ The predefined publication window extended from January 1, 2016 through September 30, 2025, and the search was executed on October 30, 2025. The complete topic search was: TS = (“remimazolam” OR “remimazolam besylate” OR “remimazolam tosilate”) AND “midazolam”. No language or document-type restriction was applied at the initial retrieval stage. The AND operator was retained to match the prespecified objective of mapping literature in which both drugs were jointly represented. This higher-specificity strategy excludes single-drug publications and is acknowledged as a study limitation (Fig. 1). The publication window was predefined to capture a recent decade of literature encompassing the expansion of remimazolam research and contemporary comparisons with midazolam.

**Figure 1.**
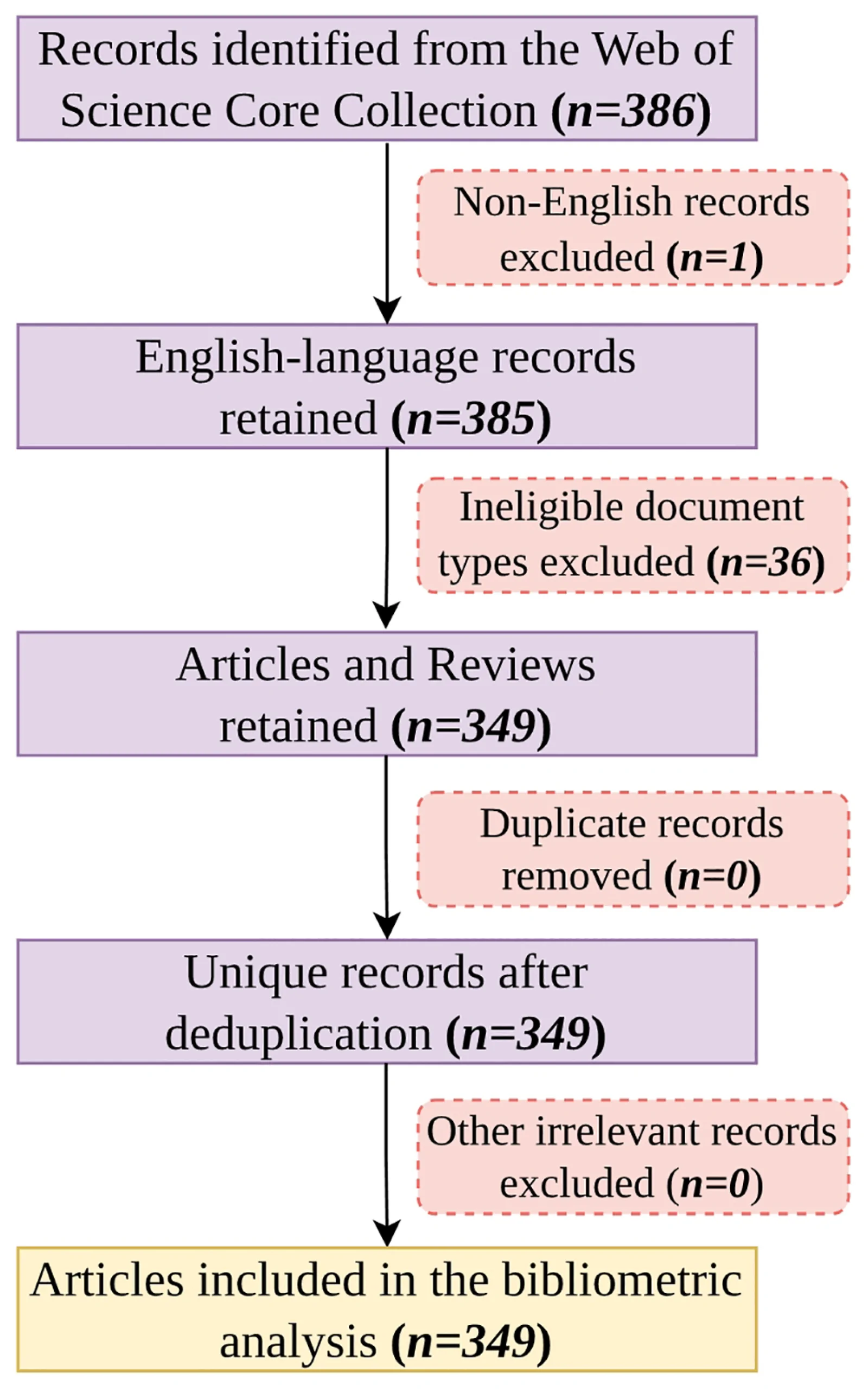
Flow diagram of literature identification and screening. The WoSCC search retrieved 386 records. A total of 37 records were excluded during language, document-type, eligibility, and duplicate screening/data cleaning, leaving 349 publications in the final bibliometric dataset. The publication window was January 1, 2016 through September 30, 2025, and the search was executed on October 30, 2025. WoSCC = Web of Science Core Collection.

### 2.2. Eligibility criteria

Records were eligible if they were original research articles or review articles; published in English; published between January 1, 2016 and September 30, 2025; and relevant to the joint remimazolam-midazolam literature, including pharmacological, clinical, or comparative topics. Records were excluded if they were retracted; clearly irrelevant to the study scope; non-English publications; or and non-article/non-review document types, including case reports, conference abstracts, editorials, letters, and news items. Eligibility criteria were applied after the initial unrestricted database retrieval.

### 2.3. Literature screening, deduplication, and data extraction

Two investigators independently screened the retrieved records, and disagreements were resolved by discussion until consensus was reached. Duplicate records were checked and removed using the duplicate-removal function in CiteSpace before the final dataset was analyzed. Self-citations were retained in the citation dataset and were not excluded from the analyses. After the application of the eligibility criteria, 349 publications were included. Bibliographic metadata, including titles, authors, affiliations, publication years, journals, abstracts, keywords, and cited references, were exported in plain-text format for analysis. The screening workflow is shown in Figure 1. Because this study analyzed bibliographic records and involved no human participants or animals, institutional ethics approval and informed consent were not required. No formal quality appraisal of individual publications was performed because the study objective was bibliometric mapping rather than clinical evidence synthesis.

### 2.4. Bibliometric analysis and visualization

CiteSpace (version 6.3.R3) was used to construct institutional collaboration networks, perform reference co-citation analysis and keyword citation-burst detection, and identify structurally important nodes. The analysis covered the predefined 2016-2025 publication window using 1-year time slices, with 2025 limited to records published through September 30. Node types were selected according to the specific analysis (Institution, Reference, or Keyword). Node selection was based on the g-index with k = 25. Pruning-related parameters were retained at the software default settings, and other parameters not specifically adjusted were likewise left at their defaults. VOSviewer (version 1.6.20) was used for keyword co-occurrence mapping and overlay visualization. Full counting and association-strength normalization were applied. Keywords occurring more than twice (minimum occurrence = 3) were eligible for inclusion in the map, while layout and visualization parameters were retained at the software default settings. Bibliometrix (version 4.0.0) in R (version 4.3.3) was used to summarize publication trends, country-, author-, and journal-level indicators, citation metrics, source impact, Bradford-law patterns, and related descriptive bibliometric measures. These tools were used complementarily to characterize productivity, collaboration, intellectual structure, and thematic evolution.^[17–20]^

## 3. Results

### 3.1. Literature screening and basic information

The initial WoSCC search yielded 386 records. After application of the English-language and document-type eligibility criteria, duplicate checking, and screening according to the predefined scope, 349 publications were included in the bibliometric dataset (Fig. 1). These publications were distributed across 137 sources and involved 1661 authors. Bibliometrix reported an annual publication growth rate of 35.84%, and the mean article age was 2.13 years (Fig. 2A). Because the 2025 dataset included only publications through September 30, the growth estimate was interpreted descriptively. The dataset contained 5963 cited references, and the average number of citations per document was 20.66. No multi-country publications were identified according to the Bibliometrix corresponding-author-country indicator (multi-country publications ratio = 0).

**Figure 2.**
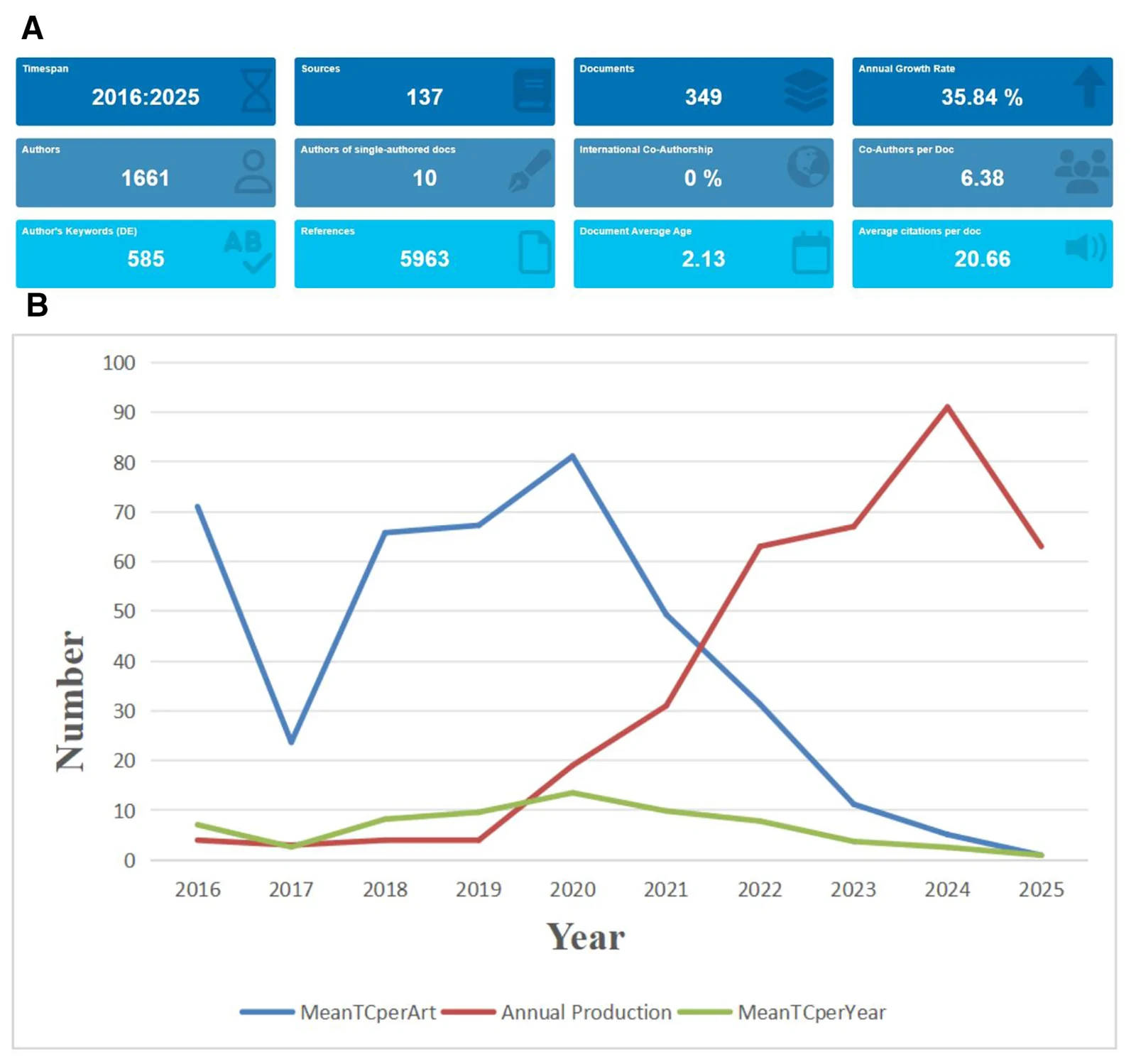
Descriptive overview and temporal trends of the bibliometric dataset. (A) Main dataset characteristics, including study period, number of sources, publications, authors, annual growth rate, cited references, citation indicators, and article age. (B) Annual publication output and citation indicators from 2016 to 2025. Data for 2025 include publications through September 30 and therefore represent a partial calendar year. MeanTCperArt, mean total citations per article; MeanTCperYear, mean total citations per year. MeanTCperArt = mean total citations per article, MeanTCperYear = mean total citations per year.

### 3.2. Publication trends and citation analysis

Annual publication output increased markedly through 2024, with particularly pronounced growth after 2020 (Fig. 2B). The lower publication count in 2025 should not be interpreted as evidence of declining research activity because the predefined publication window ended on September 30, 2025 and therefore represented only the first 9 months of that year. Mean total citations per year and mean total citations per article also varied across publication years, reflecting both changing publication volume and the different citation windows available to older and newer articles. These indicators were interpreted descriptively rather than as direct measures of study quality.

### 3.3. Analysis of countries/regions and institutions

Corresponding-author country analysis showed that China contributed the largest number of publications, followed by Japan, South Korea, and the United States (Fig. 3A). China also had the highest total citation count within the dataset (Fig. 3B). At the institutional level, Zhejiang University, PAION Deutschland GmbH, and Seoul National University were among the most productive institutions (Fig. 4B). The institutional collaboration map demonstrated multiple inter-institutional links, with several high-output institutions occupying prominent positions in the network (Fig. 4A).

**Figure 3.**
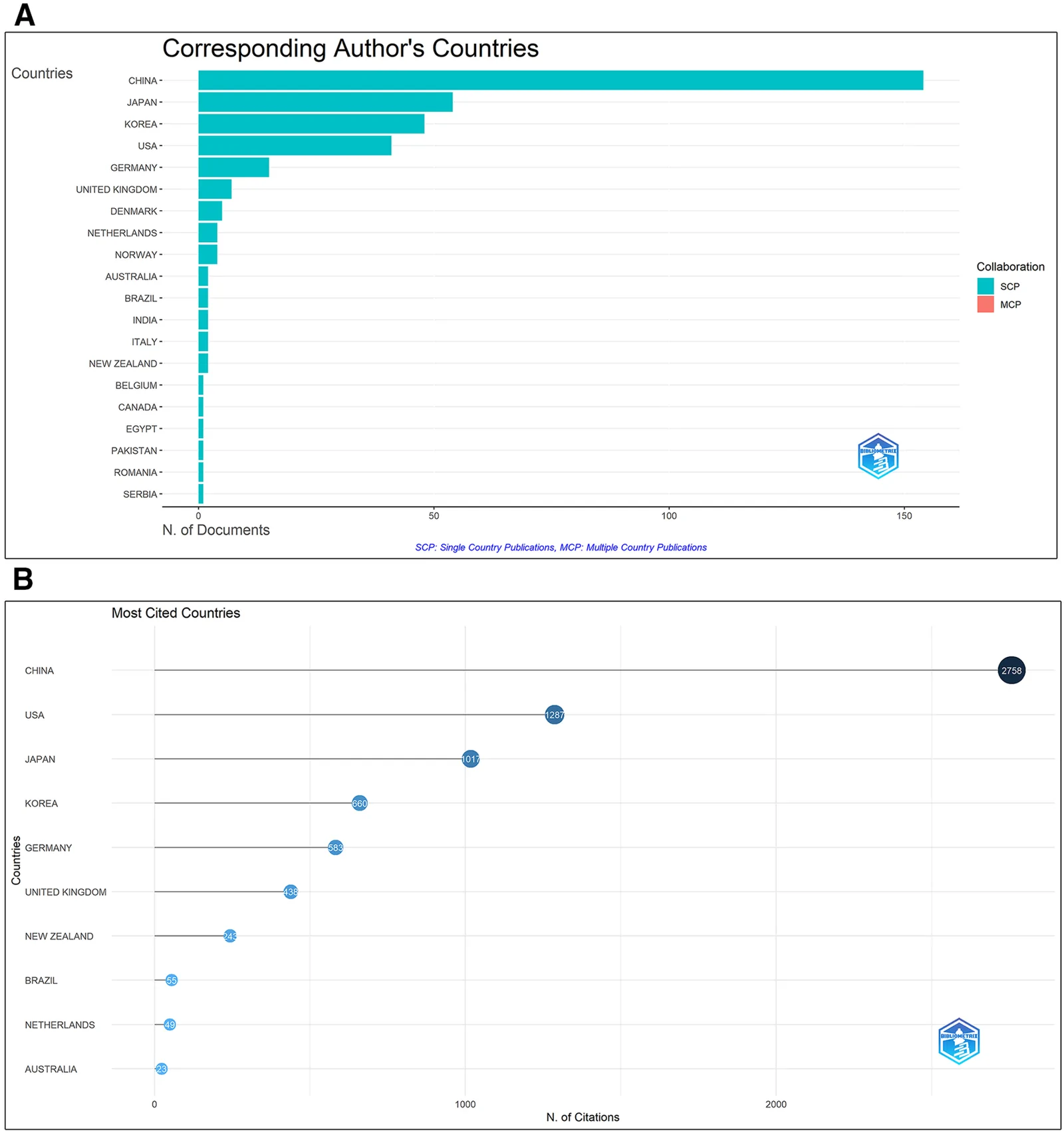
Country/region distribution of the included literature. (A) Top countries/regions by corresponding-author publication output, with single-country publications (SCP) and multi-country publications (MCP) shown where applicable. (B) Top 10 countries/regions by total citations within the dataset. MCP = multi-country publications, SCP = single-country publications.

**Figure 4.**
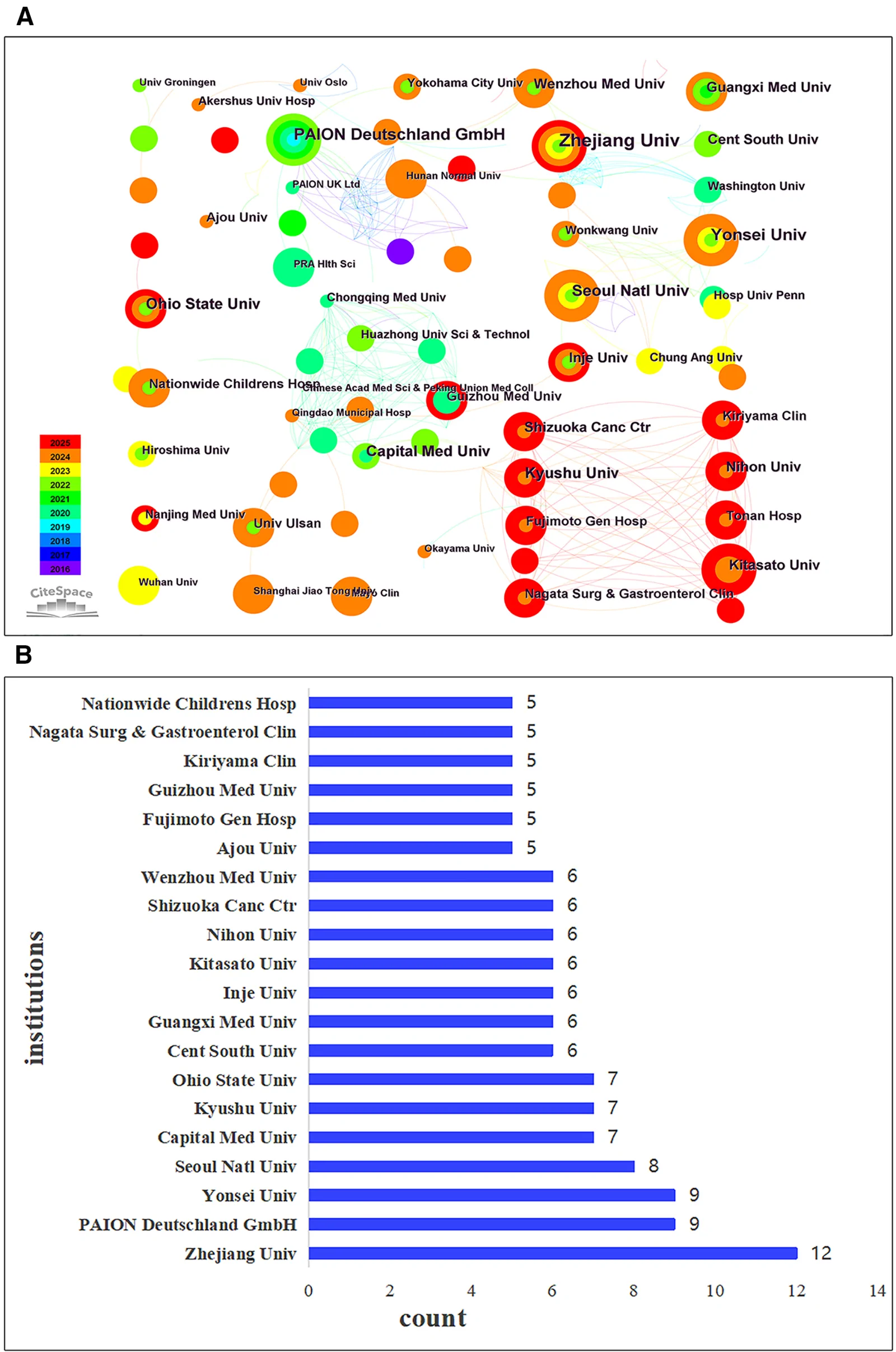
Institutional contribution and collaboration patterns in the remimazolam-midazolam literature. (A) CiteSpace-generated institutional collaboration network; nodes represent institutions and connecting lines represent co-authorship links. (B) Top 20 institutions by publication count.

### 3.4. Analysis of authors and journals

Among 1661 authors, STOEHR T had the highest publication count (15 publications), followed by SCHIPPERS F and ZHANG Y among the leading contributors (Table 2; Figure 5B). The temporal visualization showed increasing activity among several leading authors in recent years (Fig. 5A). Drug Design, Development and Therapy (27 articles) and BMC Anesthesiology (26 articles) were the 2 most productive sources in the dataset (Table 3; Figure 6A). Bradford’s law analysis further demonstrated concentration of publications within a relatively small core group of journals (Fig. 6B).

**Table 2:** Bibliometric indicators of the top 10 authors ranked by h-index in the included remimazolam-midazolam literature.

Author	h_index	g_index	m_index	TC	NP	PY_start
Stoehr T	11	15	1.57	414	15	2020
Schippers F	10	10	1.43	586	10	2019
Pesic M	7	8	1.17	162	8	2020
Goudra B	5	6	0.83	102	6	2020
Petersen Ku	5	6	0.71	221	6	2019
Tobias Jd	5	7	1.25	61	8	2022
Zhang L	5	6	1.25	125	6	2022
Zhang Y	5	9	1.00	195	9	2021
Chen J	4	4	0.80	210	4	2021
Ichijima R	4	5	1	33	7	2022

NP = number of publications, PY_start = start year of publication, TC = total citations.

**Table 3:** The top 10 journals with the most related articles.

Source	h_index	g_index	m_index	TC	NP	PY_start	JCR	IF (2024)
Bmc Anesthesiology	11	21	2.20	463	26	2021	Q2	2.60
Drug Design Development and Therapy	11	22	1.57	515	27	2019	Q2	3.00
Frontiers in Pharmacology	9	11	1.13	359	11	2018	Q1	4.80
Current Opinion in Anesthesiology	7	9	0.78	239	9	2017	Q2	2.10
Frontiers in Medicine	7	12	1.40	179	12	2021	Q1	3.00
Journal of Anesthesia	6	10	1.00	497	10	2020	Q1	2.7
Anesthesiology	5	5	0.83	358	5	2020	Q1	9.1
Journal of Clinical Anesthesia	5	6	0.83	279	6	2020	Q1	5.10
Korean Journal of Anesthesiology	5	7	1.25	158	7	2022	Q1	6.3
Minerva Anestesiologica	5	7	1.00	205	7	2021	Q2	2.8

IF = impact factor, JCR = Journal Citation Reports, NP = number of publications, PY_start = start year of publication, TC = total citations.

**Figure 5.**
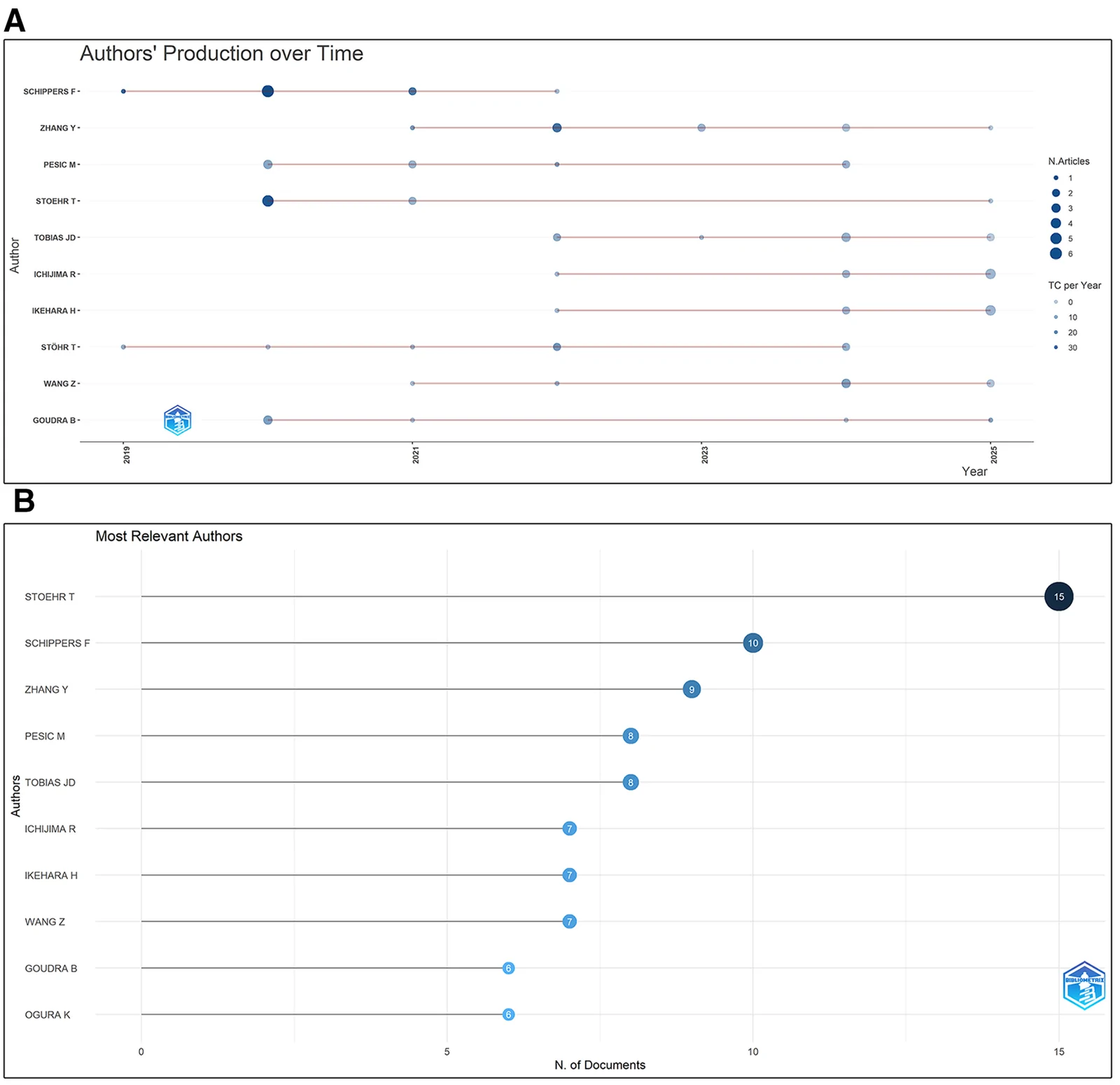
Author productivity in the included literature. (A) Production of leading authors over time; bubble size indicates the number of articles, and the accompanying citation indicator reflects citations per year. (B) Most relevant authors ranked by number of documents.

**Figure 6.**
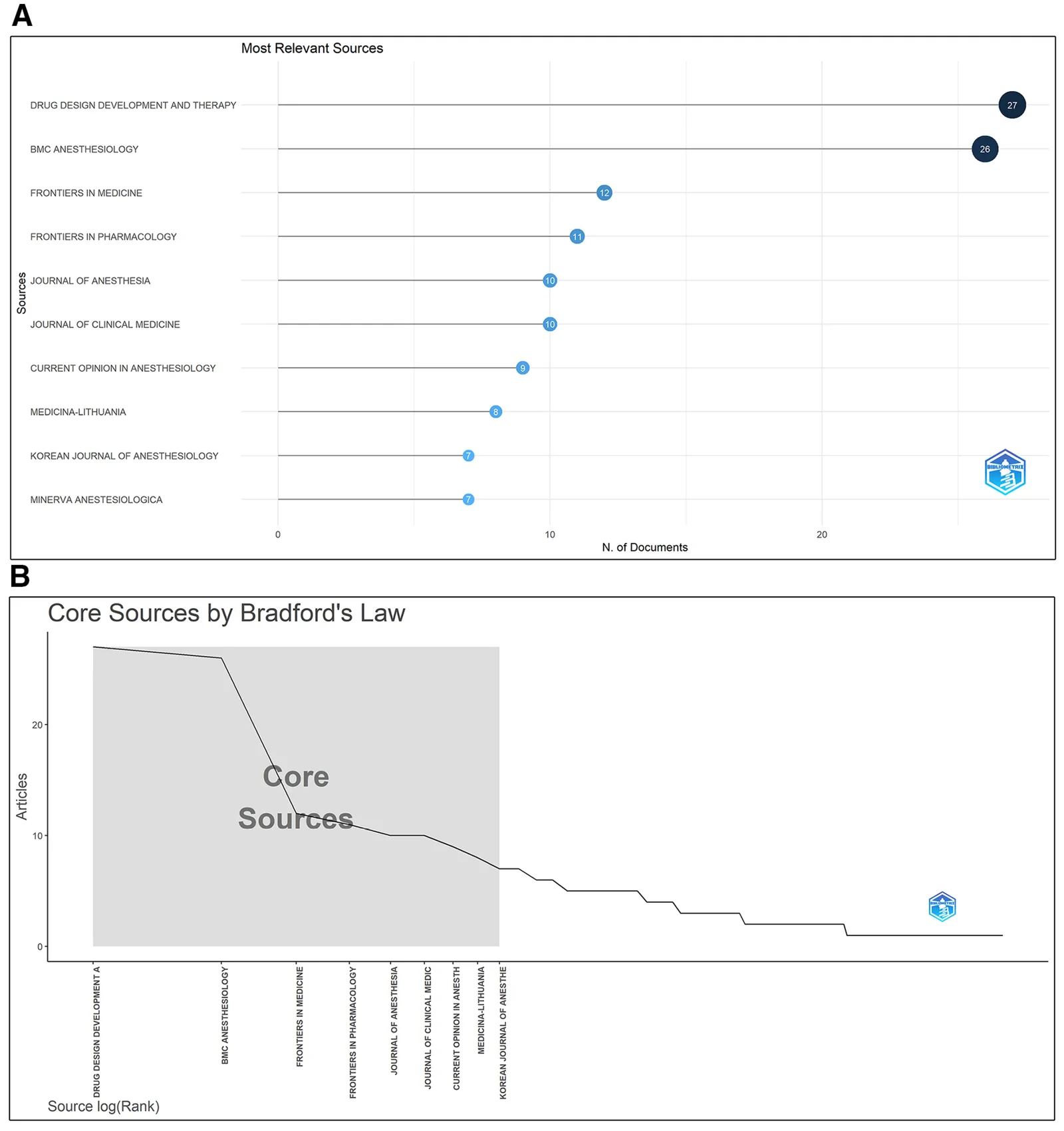
Source analysis of the included literature. (A) Most relevant sources ranked by number of included publications. (B) Core sources identified according to Bradford’s law.

### 3.5. Analysis of references and keywords

Local citation analysis identified early and frequently cited studies, including Antonik (2012) and Rex (2018), among the prominent references in the dataset (Fig. 7A). Reference publication year spectroscopy demonstrated a marked increase in cited-reference activity in more recent years (Fig. 7B). Keyword overlay analysis showed temporal shifts toward newer clinical application terms (Fig. 8A). Citation-burst analysis identified terms including “risk,” “flumazenil,” and “upper gastrointestinal endoscopy” among the recent burst keywords (Fig. 8B), indicating periods of increased research attention rather than evidence of clinical effectiveness.

**Figure 7.**
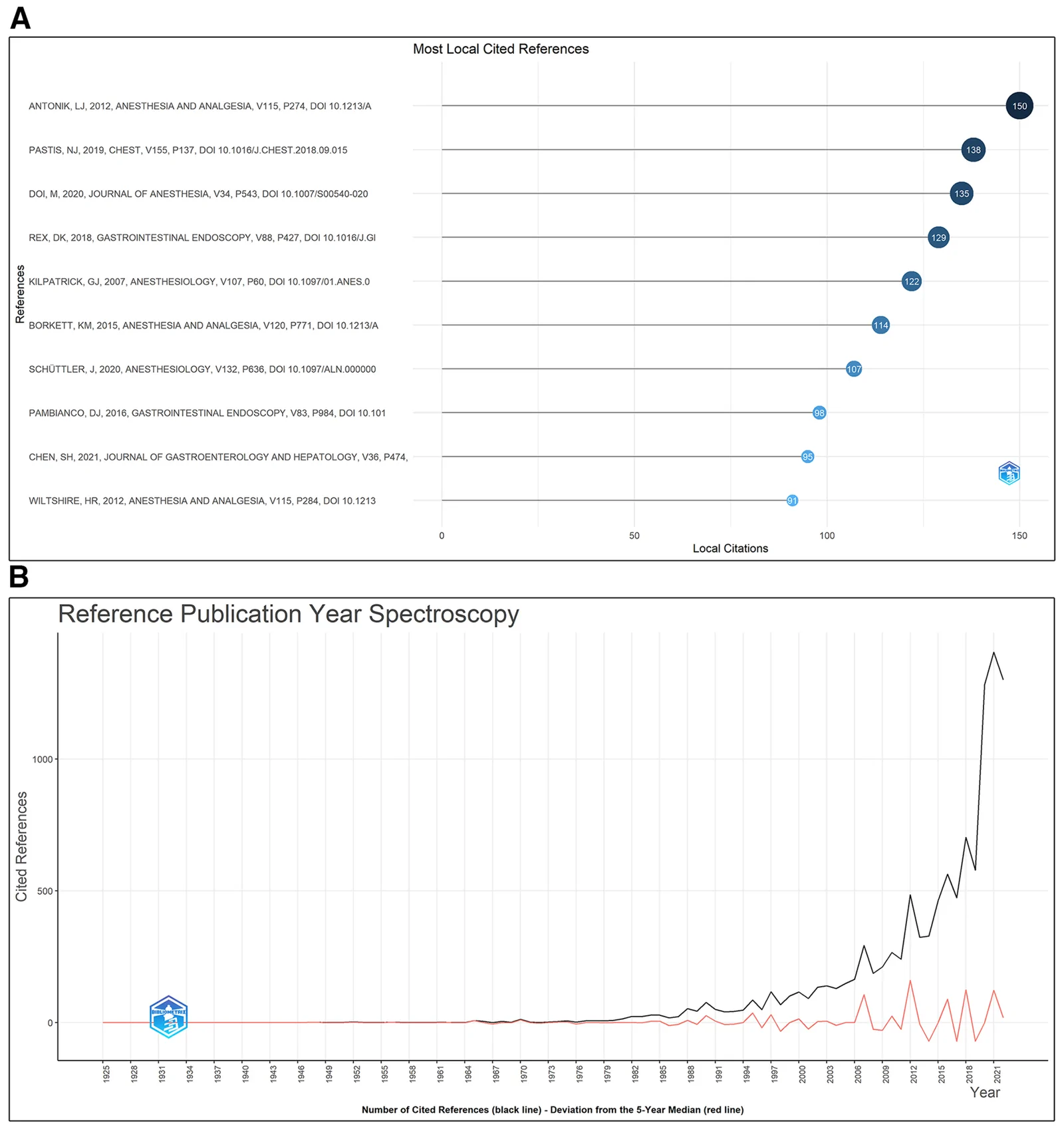
Cited-reference structure of the included literature. (A) Top 10 locally cited references within the dataset. (B) Reference publication year spectroscopy showing the temporal distribution of cited references.

**Figure 8.**
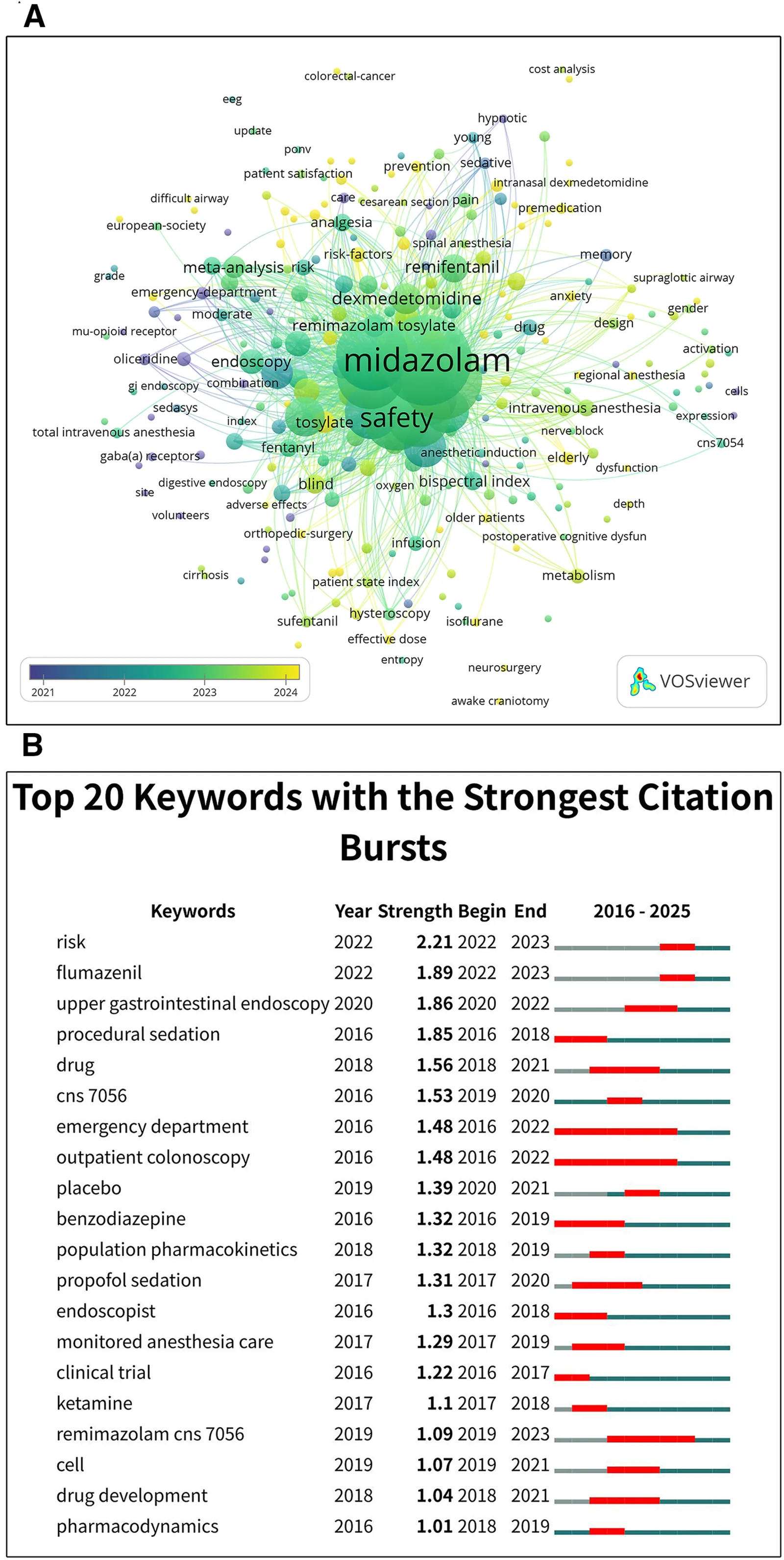
Keyword structure and temporal evolution. (A) VOSviewer overlay visualization of keyword co-occurrence. Keywords occurring more than twice (minimum occurrence = 3) were eligible for mapping; node size reflects keyword frequency, links represent co-occurrence relationships, and overlay color indicates temporal distribution. (B) CiteSpace-derived top 20 keywords with the strongest citation bursts; red segments indicate the periods during which burst activity was detected. WoSCC = Web of Science Core Collection.

## 4. Discussion

### 4.1. Summary of key findings

This bibliometric analysis describes the research landscape of publications jointly addressing remimazolam and midazolam from January 2016 through September 2025. Publication output expanded substantially and was geographically concentrated, particularly in East Asia. China contributed the largest number of publications, followed by Japan and South Korea, while the United States and several European countries also contributed to the dataset. No multi-country publications were identified by the Bibliometrix corresponding-author-country indicator, suggesting limited cross-country co-authorship within the retrieved literature. Research was concentrated in a core group of journals and institutions, and the most prominent thematic areas were pharmacokinetics, safety, efficacy, and procedural sedation. These findings map patterns of research activity and citation structure and provide context for understanding the development of the field.

### 4.2. Interpretation of major results

China accounted for the largest share of both publication output and total citations in the retrieved dataset. This pattern establishes the geographic concentration of the literature but does not, by itself, identify the reasons for that concentration. The absence of multi-country publications in the corresponding-author analysis also suggests that international co-authorship was limited within the defined dataset. Greater multinational collaboration could improve the geographic diversity of future evidence and facilitate comparisons across clinical settings and patient populations.

At the institutional level, Zhejiang University, PAION Deutschland GmbH, Seoul National University, and other high-output organizations occupied prominent positions in the collaboration network. The presence of both academic and industry-affiliated institutions reflects the translational development of the field. Publication concentration in a limited number of journals, as illustrated by Bradford’s law, further indicates that a relatively defined set of anesthesia and pharmacology sources has served as the principal dissemination base for this literature.

Keyword evolution suggests a transition from early attention to pharmacokinetic and pharmacodynamic characterization of remimazolam, including its development code CNS-7056, toward clinical application topics. Burst terms such as “upper gastrointestinal endoscopy” and “procedural sedation” indicate periods of increased research activity in specific procedural settings. More recent attention to “flumazenil” and “risk” suggests a growing emphasis on reversibility and safety-related questions. These bibliometric signals identify topics receiving attention but should not be interpreted as confirming clinical benefit or comparative superiority.

### 4.3. Comparative literature and clinical context

Comparative studies form an important component of the retrieved literature. Randomized and phase III studies have directly compared remimazolam with midazolam and/or placebo in procedural settings such as colonoscopy and bronchoscopy,^[5,6]^ helping to explain the prominence of keywords related to sedation, efficacy, safety, and gastrointestinal endoscopy. Pharmacological reviews and clinical studies have also emphasized the rapid esterase-mediated metabolism of remimazolam and its distinct pharmacokinetic profile.^[1,3,8,21]^ These clinical and pharmacological observations provide context for the bibliometric patterns identified in this study and help explain the prominence of efficacy, safety, and procedural-sedation themes.

Interest in specific populations is also evident in the broader remimazolam literature. Pharmacokinetic evaluation in hepatic and renal impairment^[4]^ and studies involving older patients^[22,23]^ illustrate the growing attention to patient-specific drug handling and recovery characteristics. These studies point to patient-specific pharmacokinetics and recovery as important directions for future direct comparative studies and evidence synthesis using clinically meaningful outcomes.

### 4.4. Future research directions

Future research should prioritize well-designed head-to-head studies that directly compare remimazolam and midazolam across clearly defined procedural and anesthesia settings. Multicenter and multinational designs would improve generalizability and address the limited international co-authorship observed in the present dataset. Research should also distinguish clinically meaningful outcomes such as hemodynamic instability, respiratory events, recovery time, delirium, patient satisfaction, and resource utilization rather than relying on broad claims of efficacy or safety. In parallel, model-informed precision dosing studies may help clarify how patient-specific factors influence remimazolam exposure and response.^[24,25]^

Additional work in older adults and patients with hepatic or renal dysfunction is warranted because these populations are frequently clinically relevant and have been represented in pharmacokinetic or comparative studies.^[4,22,23]^ Similar caution should be applied when extending findings to other special populations for which the available evidence base may be smaller. Future studies should therefore define the target population, comparator, dosing strategy, and clinically important outcomes a priori and report adverse events and recovery endpoints consistently.

### 4.5. Strengths and limitations

This study has several strengths, including a predefined bibliometric workflow, independent screening by 2 investigators, and the complementary use of CiteSpace, VOSviewer, and Bibliometrix to characterize publication output, collaboration, citation structure, and keyword evolution.

Several limitations should be considered. First, only WoSCC was used. Although this provided a standardized citation dataset, relevant records indexed exclusively in Scopus, PubMed/MEDLINE, Embase, or other databases may have been missed, which can introduce database-selection bias. Second, the final analysis was restricted to English-language articles and reviews, potentially underrepresenting research published in other languages. Third, the intentionally specific AND-based search required joint representation of remimazolam and midazolam; consequently, remimazolam-only and midazolam-only studies were outside the prespecified bibliometric scope, and the findings should not be interpreted as a complete map of either drug’s entire literature. Fourth, citation-based indicators are influenced by publication age, database coverage, and retained self-citations. Because self-citations were not excluded, some citation counts and network metrics may have been affected. Fifth, the publication window ended on September 30, 2025, so the 2025 data represent only the first 9 months of the calendar year. Although the search was executed on October 30, 2025, allowing an additional month for database indexing, residual indexing delays may still have affected recently published records. Finally, bibliometric methods describe patterns of research activity and scholarly influence and do not directly estimate comparative treatment effects.

## 5. Conclusion

This bibliometric analysis maps the literature jointly addressing remimazolam and midazolam from January 2016 through September 2025. Research activity expanded markedly and was geographically concentrated in East Asia, with major themes centered on pharmacokinetics, safety, efficacy, and procedural sedation. Collaboration and citation mapping identified a relatively concentrated group of countries, institutions, authors, and journals, while keyword evolution highlighted increasing attention to clinical applications, flumazenil, and safety-related topics. Together, these findings provide a structured overview of the field’s development and identify areas where stronger collaborative and comparative evidence is still needed.

Future research should prioritize multinational and multicenter comparative studies, transparent reporting of clinically meaningful outcomes, and patient-specific dosing research. Broader database strategies and complementary systematic reviews or meta-analyses will also be important for evaluating clinical effectiveness beyond the descriptive scope of bibliometric analysis.

## Author contributions

**Conceptualization:** Shengzhen Luo, Yongwang Li.

**Funding acquisition:** Liang Cong, Shengzhen Luo, Yongwang Li.

**Data curation:** Yu Liu, Liang Cong, Yuying Ma, Yongwang Li.

**Investigation:** Liang Cong, Hui Xiao.

**Methodology:** Yu Liu, Shengzhen Luo, Yuying Ma.

**Software:** Yu Liu, Hui Xiao, Yongwang Li.

**Supervision:** Yu Liu, Yongwang Li.

**Writing – original draft:** Yu Liu, Shengzhen Luo, Yuying Ma, Yongwang Li.

**Writing – review & editing:** Liang Cong, Hui Xiao, Yongwang Li.
